# Domestication and Temperature Modulate Gene Expression Signatures and Growth in the Australasian Snapper *Chrysophrys auratus*

**DOI:** 10.1534/g3.118.200647

**Published:** 2018-12-27

**Authors:** Maren Wellenreuther, Jérémy Le Luyer, Denham Cook, Peter A. Ritchie, Louis Bernatchez

**Affiliations:** * The New Zealand Institute for Plant and Food Research Limited, Nelson, New Zealand; † School of Biological Sciences, The University of Auckland, New Zealand; ‡ Institut de Biologie Intégrative et des Systèmes (IBIS), Pavillon Charles-Eugène Marchand, Université Laval, Québec, Canada; § Ifremer, UMR 241 Ecosystèmes Insulaires Océaniens, Centre Ifremer du Pacifique, BP 49, 98725 Tahiti, Polynésie française; ** School of Biological Sciences, Victoria University of Wellington, Wellington, New Zealand

**Keywords:** Domestication, Temperature, Transcriptomics, Growth, Sparidae

## Abstract

Identifying genes and pathways involved in domestication is critical to understand how species change in response to human-induced selection pressures, such as increased temperatures. Given the profound influence of temperature on fish metabolism and organismal performance, a comparison of how temperature affects wild and domestic strains of snapper is an important question to address. We experimentally manipulated temperature conditions for F_1_-hatchery and wild Australasian snapper (*Chrysophrys auratus*) for 18 days to mimic seasonal extremes and measured differences in growth, white muscle RNA transcription and hematological parameters. Over 2.2 Gb paired-end reads were assembled *de novo* for a total set of 33,017 transcripts (N50 = 2,804). We found pronounced growth and gene expression differences between wild and domesticated individuals related to global developmental and immune pathways. Temperature-modulated growth responses were linked to major pathways affecting metabolism, cell regulation and signaling. This study is the first step toward gaining an understanding of the changes occurring in the early stages of domestication, and the mechanisms underlying thermal adaptation and associated growth in poikilothermic vertebrates. Our study further provides the first transcriptome resources for studying biological questions in this non-model fish species.

The domestication of plants and animals marks a major evolutionary transition and ascertaining the molecular and physiological basis of domestication and breeding represents an exciting area of interdisciplinary research (Zeder 2015). Compared with terrestrial animals, the domestication of fish for human consumption started only recently (Yáñez *et al.* 2015) and with the exception of a few species, such as the common carp (*Cyprinus carpio*) or Nile tilapia (*Oreochromis niloticus*), most domestication efforts date back to the early 1980s (Balon 2004). Consequently, most cultured fish species have changed only slightly from their wild conspecifics compared to other organisms (Olesen *et al.* 2003; Li and Ponzoni 2015). This represents a unique opportunity to study how animals evolve during the transition from wild to captive conditions, as well as during the first generations of domestication.

For poikilothermic species such as fish, temperature plays a profound and controlling role in their biological functioning (Fry 1971; Hochachka and Somero 2002). Affecting cellular components and molecular functions via, for instance, a change in the viscosity of body fluids, fluidity of cell membranes, and enzyme kinetics (Currie and Schulte 2014), temperature influences the pathways by which individuals allocate energy to competing functions (Claireaux and Lefrançois 2007; Khan *et al.* 2015). For eurythermal fish (which can survive across a broad temperature range; (Logan and Buckley 2015), such as the Australasian snapper (*Chrysophrys auratus*, Sparidae), environmental fluctuations dictate that their body temperatures vary in both space and time. When environmental temperatures are within a ‘zone of tolerance’, physiological, biochemical and behavioral aspects of the organism’s biology are at, or near, optimal (Pörtner 2010; Currie and Schulte 2014). Yet when temperatures are at the extremities of this tolerable range both acute and chronic stress responses can be observed; that translate into reduced organismal performance, adversely affecting growth, routine activity, or reproduction (Fry 1971; Mininni *et al.* 2014; Schulte 2014). However, the thermal tolerances of fish commonly show significant plasticity, with notable intraspecific variability and acclimation responses reported in both eurythermal and stenothermal (narrow thermal tolerance) species (Seebacher *et al.* 2005; Anttila *et al.* 2013; Sandblom *et al.* 2014; Metzger and Schulte 2018). Given the significant influence of temperature on fish metabolism and organismal performance, a comparison of how temperature affects wild and domestic strains of snapper is an important question to address.

Rapid growth is a key determinant of commercial farming success, and is heavily modulated by the ambient temperature (Mininni *et al.* 2014; Bizuayehu *et al.* 2015; Besson *et al.* 2016). Moreover, growth is frequently correlated with a number of life-history traits, such as gonad maturation and reproductive timing (Schaffer 1979; Thorpe 1994; Devlin and Nagahama 2002). Consistent with the complex associations of growth with other traits is the finding that the genetic architecture of this trait is typically polygenic and determined by complex genes networks (Filteau *et al.* 2013; Wellenreuther and Hansson 2016). This complexity makes it challenging to understand the specific genetic and physiological determinants which underpin faster growing phenotypes that need to be targeted when selectively breeding for enhanced growth.

For many commercially valuable species, selective breeding programs have been initiated to produce strains that have an improved tolerance to domestic conditions and develop more quickly into a marketable phenotype (Olesen *et al.* 2003; Bernatchez *et al.* 2017). Understanding how domesticated organisms have been transformed from their ancestral wild type is valuable both from a genetic and evolutionary perspective, and provides fundamental information for future enhancement of strains through selective breeding. Genetic differences between wild and domesticated individuals can arise in three main ways. First, they can be an inevitable by-product from a relaxation of natural selection pressures in captive conditions (Hutchings and Fraser 2008). By virtue of being raised in an artificial and controlled setting, farmed hatchery populations undergo inadvertent genetic changes because fish experience no natural predator nor significant foraging challenges. Second, intentional domestication selection (*e.g.*, to enhance commercially relevant traits) and inadvertent or novel natural selection (*e.g.*, the amount and quality of space provided) tend to favor fish that survive best in domesticated conditions, which can affect linked characteristics. Third, the often small population sizes of farmed species can cause strong genetic drift and inbreeding, which can reduce the level of genetic diversity, increase the frequency of deleterious alleles, or overwhelm the strength of artificial selection and eliminate commercially important variation (Taberlet *et al.* 2011). This third process is commonly observed in the rapid loss of standing genetic variation in domesticated strains following a few generations of selective breeding (Hill *et al.* 2000).

Due to the recent domestication history of many fish species, they provide an ideal model for investigating the genetic changes associated with domestication and captive breeding because there are still natural populations that can be used as a reference (Mignon-Grasteau *et al.* 2005). Several studies to date have investigated the effects of domestication on fish gene expression patterns (Roberge *et al.* 2005; Devlin *et al.* 2009; Sauvage *et al.* 2010), but these are almost entirely focused on salmonid fishes. Moreover, much less work has been conducted to dissect the more specific changes responsible for accelerated growth in selectively bred strains. Indeed, while the factors and pathways underlying differential growth in mammals have been established in considerable detail, knowledge of the relevant genes involved in growth variation in fish is much more limited (Fuentes *et al.* 2013). This is due to the relative lack of fish-specific molecular tools and functional studies, and compounded by the extra level of complexity in fish genomes due to whole genome duplication and the subsequent rediploidisation event(s) (Volff 2005).

Here we explore temperature-induced growth and stress responses in wild and domesticated strains of the Australasian snapper *Chrysophrys auratus* to gain a better understanding of the domestication process and identify the genes and pathways important for growth in teleost fish. This marine species has a distribution from 25 to 40° S in temperate and sub-tropical waters in New Zealand and Australia and is highly valued by commercial and recreational fisheries (Parsons *et al.* 2014). Despite its commercial and recreational importance, no transcriptomic studies have been performed on this species so far. We performed a manipulative experiment and held wild and first generation domesticated snapper strain in cold and warm treatments and measured their growth and white muscle RNA expression profiles. The domesticated snapper strain was obtained from wild hatchery housed broodstock parents, *i.e.*, gone through one generation of selection, whereas the wild strain was collected from a wild population prior to the experiment. First, we compared phenotypic responses of wild and domesticated snapper to the temperature treatments to quantify any growth-related changes. Second, we compared gene expression profiles to identify the genes that are affected by genotype (wild *vs.* domesticated) and temperature, and their interaction. Finally, we used co-expression network models to gain insights into the metabolic modules and pathways affected in these genotype-specific temperature responses, to better understand the growth and metabolic pathways in this species.

## Materials and Methods

### Fish holding and experimental setup

The New Zealand Institute for Plant and Food Research (PFR) has been breeding *C. auratus* since 2004 in Nelson, and in 2014 a selective breeding program was started to select for enhanced growth in this species. Experiments were carried out on 2-year-old wild-caught and hatchery reared domesticated *C. auratus*, from December 2015 to January 2016 at the PFR Seafood Facility in Nelson. Wild *C. auratus* were captured by trawl during a research voyage of the vessel FV Bacchante in the inner Tasman Bay (centered on S41 12.700 E173 09.400) in February 2015 and transferred to the PFR facility the same day (and thus had time to acclimatise in the hatchery for at least 10 months prior to the trial). The domesticated *C. auratus* strain was raised from naturally spawned larvae propagated from wild-caught broodstock held at the PFR Nelson Seafood Research Facility. Prior to experimentation both populations were acclimatised in separate 5000-L tanks provided with flow-through filtered seawater at ambient temperatures and light conditions. Both wild and domesticated *C. auratus* were fed on equivalent rations of mixed fish pieces, commercial aquaculture pellet (3 mm, Nova ME; Skretting, Cambridge, Tas., Australia) and an in-house formulated gel diet consisting of 21.3% protein, 2.7% lipid, 5.6% carbohydrate, 7.7% ash, and 62.7% moisture. All rearing, holding and sampling procedures were performed using standard hatchery practices in accordance with New Zealand’s Animal Welfare Act (1999).

Twenty wild-caught fish and 20 hatchery reared fish (referred to herein as genotypes) were weighed, measured and imaged, under anesthesia (20ppm AQUI-S, AQUIS NZ Ltd, Lower Hutt, New Zealand), and moved into four 800-L tanks supplied with 1-µm filtered, UV-sterilized, temperature-controlled flow through seawater (35 ppt salinity, ambient temperature = 17.0°). Each tank consisted of either 10 wild-caught or 10 domesticated *C. auratus* individuals.

Different thermal regimes were generated following a 5-day period of acclimation time at a nominal temperature of 17.0°. One tank of the wild-caught fish and one of the domesticated strain were exposed to a temperature decrease of 1.0° day^-1^ while the remaining wild and domesticated strain were exposed to a temperature increase of 1.0° day^-1^. Following the 5-day period of increased/decreased temperatures the desired temperature differential of either 13.0 or 21.0° was established, henceforth referred to as low and high temperature treatments, respectively. These temperatures were chosen to reflect seasonal differences that these strains would experience in their local environment in Nelson and Tasman Bay (*i.e.*, winter and summer temperatures). Temperature loggers (HOBO, Onset Computer Corporation, MA, USA) showed that the thermal environment for the duration of the experiment maintained the desired treatment temperatures, with the low treatment having a mean temperature of 13.8° and the high treatment having a mean temperature of 21.9°, with minimal variation across the experiment (absolute maximum differences in the low and high treatments were ±1.7° and ±0.5°, respectively).

Throughout the experiment, fish were maintained solely on the commercial pelletized (Skretting) diet described above at a ration equivalent to 2% body-mass per day, provided on a daily basis. Dissolved oxygen levels were checked multiple times per day to ensure levels were kept at >90% and tanks were cleaned every 3–4 days. Once the desired temperatures were reached, the experiment was allowed to run for 18 days.

### Animal sampling, RNA extraction and sequencing

Upon termination of the experiment, eight fish from each of the four treatments (genotype x temperature treatment) were anesthetized (25 ppm AQUI-S) then netted from their tank and subsequently euthanised with an overdose of anesthetic (60ppm AQUI-S). Immediately following euthanasia, fish were imaged and weighed for identification and phenotyping (Table 1). Then a 2- to 3-mL sample of mixed whole blood was collected from the caudal vein and placed on ice using 21g hypodermic needles and EDTA-treated vacutainers (BD, Franklin Lakes, NJ, USA). For the transcriptome assembly, 12 brain and 12 epaxial white muscle tissue samples (removed from the D-muscle block immediately anterior of the dorsal fin), as well as three whole larvae (2–3 months of age) were preserved in RNAlater (Ambion, USA) at 4° overnight before being transferred to −80° for long-term storage.

**Table 1:** Phenotypic values of wild and domesticated *C. auratus* used in the low and high temperature treatments. Errors in brackets are standard error of the mean, n = 8. All physiological traits were measured upon termination of the experiment

	Low Temperature Treatment		High Temperature Treatment	
	Wild Population	Domesticated Population	Wild Population	Domesticated Population
**Starting mass (g)**	159.5 (6.7)	149.5 (4.7)	145.9 (7.9)	151.2 (4.6)†
**Terminal mass (g)**	144.3 (7.0)	150.5 (4.8)	154.4 (7.0)*	178.9 (6.6) †*
**Starting fork length (mm)**	189.6 (3.1)	194.2 (1.6)	186.9 (3.4)	194.7 (3.3)†
**Terminal fork length (mm)**	189.6 (3.1)	194.4 (1.6)	190.3 (3.0)	200.3 (3.0)
**SGR (% Body mass day-1)**	−0.37 (0.09)	0.03 (0.05)†	0.22 (0.07)*	0.60 (0.07)†*
**LGR (mm day-1)**	0.01 (0.00)	0.01 (0.01)	0.12 (0.03)*	0.21 (0.05) †*
**Hepatosomatic index (HSI)**	1.57 (0.13)	1.48 (0.13)	1.57 (0.19)	1.67 (0.10)
**Cardiosomatic index (CSI)**	0.15 (0.03)	0.12 (0.03)	0.09 (0.01)	0.12 (0.01)†
**Haematocrit (%)**	28.0 (1.0)	33.4 (2.2)	30.4 (0.5)*	32.6 (1.0)
**Haemoglobin (g dL-1)**	6.0 (0.1)	4.8 (0.4)†	6.2 (0.2)	6.2 (0.2)
**MCHC (g dL-1)**	21.4 (0.7)	14.4 (0.5)†	20.5 (3.5)	19.0 (0.7)*
**Triglycerides (g dL-1)**	1.00 (0.10)	2.50 (0.29)†	4.24 (0.52)*	4.13 (0.21)*
**Plasma lactate (mM)**	2.23 (0.38)	1.68 (0.10)	3.19 (0.30)	3.45 (0.28)*
**Plasma glucose (mM)**	11.33 (0.69)	11.76 (1.01)	9.22 (0.58)*	6.47 (0.60)†*

* denotes significantly different values between temperature treatments within the same (wild/domesticated) population, † denotes significant differences between wild and domesticated *C. auratus* at comparable temperatures. Analyses were performed using parametric ANOVA with a Bonferroni adjustment. Significance was accepted at *P* < 0.05, non-parametric data were log transformed to meet assumptions of normality or homoscedasticity. LGR = linear growth rate; MCHC = mean corpuscular hemoglobin concentration; SGR = specific growth rate.

Haematological parameters were assessed in all individuals to compare the physiological conditions between the two treatments. Haematocrit (Hct) was measured from whole blood immediately after collection using 75-mm capillary tubes spun at 23,020 g, (Hettich Universal 320R with a 1650 rotor Heidelberg, Germany at 4°, for 5 min,). Haemoglobin concentration [Hb] was then measured spectrophotometrically by mixing 10 µL of whole blood into modified Drabkin’s reagent, measured in 1-mL cuvettes (Wells *et al.* 2007). Mean corpuscular hemoglobin concentration (MCHC) was estimated from the ratio of ([Hb]/Hct). Plasma was then separated by centrifugation (3230 g, Hettich Universal 320R with a 1324 rotor with 1486 inserts Heidelberg, Germany at 4°, for 10 min) in 200-uL aliquots, snap frozen in liquid nitrogen and then stored at −80° for later analysis of plasma metabolites. Plasma osmolarity was determined from freeze-thawed plasma samples using a Wescor vapor pressure osmometer (Vapro 5520, Wesco Inc. UT, USA). Plasma triglycerides were determined on a clinical blood analyzer (Reflotron, Roche, Germany) using standard methods. Plasma lactate and glucose were measured using commercially available enzymatic assays kits (Megazyme K-Late and K-Gluc, Food Tech Solutions, New Zealand), with the assays performed and analyzed in a 96-well microplate format (Clariostar, BMG Labtech, Germany).

Total RNA was extracted from 12 fish (three fish from each treatment: wild snapper x high and low temperature; domesticated snapper x high and low temperature, see Table 2) using the Trizol LS Reagent (Life Technologies) according to manufacturer’s instructions. RNA samples were individually prepared (including mRNA enrichment with a poly(A) method) for sequencing using the Illumina Tru-Seq kit on two lanes of an Illumina HiSeq 2000 sequencer (paired-end 100-bp sequencing, 160-bp insert length, see Supplementary Table S1) at the Beijing Genomics Institute (BGI), Shenzhen, China. Prior to sequencing each RNA sample was quality checked by BGI to ensure sufficient sample quality and quantity.

**Table 2:** Sequencing results, reads pre-processing and mapping summaries

Treatment	Samples	Raw PE reads	Trimmed PE reads	Mapped PE reads	Mapping rate (%)
Domesticated - High	DH.1MA	23.0 M	22.4 M	18.0 M	80.1
Domesticated - High	DH.4MA	23.3 M	22.7 M	18.2 M	80.4
Domesticated - High	DH.5MA	33.9 M	21.5 M	17.6 M	81.9
Domesticated – Low	DL.4MA	23.3 M	22.6 M	17.9 M	79.3
Domesticated – Low	DL.5MA	23. 4M	22.8 M	17.9 M	78.6
Domesticated – Low	DL.6MA	23.4 M	22.8 M	17.7 M	77.9
Wild –High	WH.1MA	22.3 M	21.6 M	17.6 M	82.0
Wild –High	WH.2MA	22.5 M	21.9 M	18.0 M	81.9
Wild –Low	WL.1MA	22.9 M	22.2 M	15.9 M	71.6
Wild –Low	WL.3MA	23.2 M	22.6 M	17.6 M	77.8
Wild –Low	WL.6MA	22.3 M	21.6 M	16.4 M	75.7

### Sequence data processing and de novo transcriptome assembly

All samples were used for the transcriptome assembly, but only the white muscle samples from the temperature experiment were used for the gene expression study. Sequences were first quality trimmed (trailing: 20; lowest quality: 30) and cropped for minimum length (< 60 bp) using Trimmomatic v0.36 software (Bolger *et al.* 2014). Trimming also included removal of putative contaminants from the UniVec database (https://www.ncbi.nlm.nih.gov/tools/vecscreen/univec/). Trimmed sequences were further quality checked with FastQC v0.11.5 software (https://www.bioinformatics.babraham.ac.uk/projects/fastqc/) based on per base and per sequences qualities scores, overrepresented sequences and adapter content metrics. At this step, one individual was removed because of lower sequencing quality (Supplementary Table S1, individual removed WH3).

Paired-end reads were assembled into transcripts (minimum length 200 bp) using the Trinity v2.2.0 *de novo* assembly pipeline (Haas *et al.* 2013) with a default k-mer size of 25 bp. Raw transcripts (n = 242,320) were filtered for the presence of Open Reading Frames (ORFs) (length ≥ 300 nt), longest isoform matches and mapping rate (≥ 1 TPM), following the methodology used by Pasquier *et al.* (2016) (Pasquier *et al.* 2016) Mapping for transcriptome filtering was conducted with Bowtie2 v 2.3.4.1 (Langmead and Salzberg 2012) and using RSEM count estimates (Li and Dewey 2011). The remaining transcript sequences were searched against the Uniprot-Swissprot database (blastX; e-value < 10e-6). While searching for comparisons across multiple species might not be relevant for genes that have lost their function across evolutionary times, it represents one of the most effective and powerful approaches to assess global functional annotation for *de novo* references (Zhou and Gibson 2004; Pasquier *et al.* 2016; Carruthers *et al.* 2018). For quality checks, the *de novo* transcriptome completeness was assessed with the BUSCO v1.1b metazoa database (Simão *et al.* 2015). We used TransRate v1.0.3 quality statistics to validate each transcriptome filtering step (Smith-Unna *et al.* 2016).

### Differential gene expression and genotype × environment interaction

We first investigated the genotype × environment interaction (GEI) from gene expression levels using a generalized linear model (GLM) approach (temperature × genotype) with the ‘*glmFit*’ function and a likelihood-ratio test implemented in the R package edger (Robinson *et al.* 2010). We only considered genes with a false discovery rate (FDR) < 0.01 to be significant. To further quantify the additive effect of temperature and genotype, we conducted a GLM approach (temperature + genotype) on the dataset with prior removal of differentially genes with a significant interaction term. Genes were considered significantly expressed when FDR < 0.01 and |logFC| ≥ 2 (*e.g.*, a fourfold difference between treatments).

### Co-expression network analysis

Signed co-expression networks were built using the R package weighted gene co-expression network analysis (WGCNA) following the protocol proposed by Langfelder and Horvath (Langfelder and Horvath 2008) based on normalized log-transformed expressions values. The main goal of this analysis was to cluster genes in modules associated with genotype and temperature effects and relevant gradients of traits under investigation. Briefly, we fixed a soft threshold power of 22 using the scale-free topology criterion to reach a model fit (|R|) of 0.81. The modules were defined using the ‘*cutreeDynamic*’ function (minimum 30 genes by module and default cutting-height = 0.99) based on the topological overlap matrix and, a module Eigengene distance threshold of 0.25 was used to merge highly similar modules. For each module we defined the module membership (kME, correlation between module Eigengene value and gene expression values). Only modules with an absolute Pearson’s correlation value (|R|) > 0.75 with temperature and genotype factors and with p-value < 0.001 were conserved for downstream functional analysis. For module visualization we selected the top 30 genes (hereafter called hub genes) based on the kME values. The resulting gene networks were plotted with Cytoscape v3.5.1 (Shannon *et al.* 2003).

### Gene ontology and KEGG pathway visualization

Gene ontology enrichment analyses were conducted using GOAtools v0.6.5 (Klopfenstein *et al.* 2018) based on the go-basic database (release 2017-04-14). Our background list included genes used for the gene network construction (after removing low-expressed genes, n = 13,282; see section above). Only gene ontology (GO) terms with p-adj < 0.05 and including at least three genes were considered (Supplementary Table S2). We matched each differentially expressed gene to the corresponding Kyoto Encyclopedia of Genes and Genomes (KEGG) pathway via the online web server KAAS (Moriya *et al.* 2007). The respective KEGG pathways were plotted using the Pathview R package (Luo and Brouwer 2013).

### Data availability

The raw data can be accessed via a data repository hosted by the national infrastructure platform Genomics Aotearoa in New Zealand (https://www.genomics-aotearoa.org.nz/data). For reproducibility, the codes are deposited in GitHub (https://github.com/jleluyer/PFR_snapper). Supplemental material available at Figshare: https://doi.org/10.25387/g3.7388714.

## Results and Discussion

### Phenotypic changes

**Figure 1:**
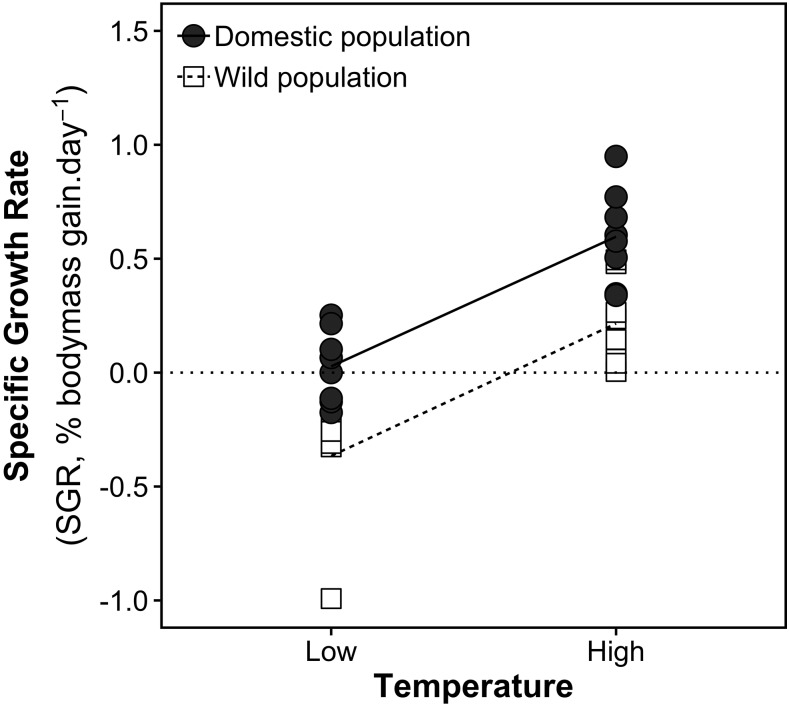
Specific growth rates of wild and domesticated *C. auratus* (8n/treatment) at the start and end of the low and warm temperature experiment.

Upon termination of the experiments, marked phenotypic differences were observed between genotypes and temperature treatments (Table 1, Figure 1). At the beginning of the trial we measured slight and non-significant differences in starting mass and length between the wild and domestic *C. auratus*. At the end of the trial, mass and mass length were significantly higher for the domestic genotype (Table 1). Temperature-related growth differences were also visible with both genotypes achieving significantly larger growth (length and mass) gains in the high temperature treatment (Table 1). Moreover, low temperature had a profound effect on growth rate, with a net effect of near-zero growth in the domestic strain and negative growth (a reduction in mass) in the wild strain. None or negative growth is probably associated with a limited metabolic capacity due to reduced aerobic metabolic scope (Pörtner 2010). Growth differences may also indicate that the domesticated strain is more resistant to cold stress than the wild strain, but also is more responsive to increases in temperature, each of which benefits maintenance and growth respectively.

### Transcriptome assembly quality and completeness

We used a total of 2.2 Gb paired-end reads to assemble a raw transcriptome containing 242,320 transcripts (215 Mb). The final reference assembly (after filtering) based on the different tissues types and replicate individuals represents a set of 33,017 transcripts (N50 = 2,804; GC content = 48.6%; Table 3). The final transcriptome completeness evaluation with BUSCO v1.1b (Simão *et al.* 2015) indicated that 96% of the highly-conserved single-copy metazoan genes (n = 978) were present in the transcriptome sequence (including 89.5% complete and present as a single-copy). Transcriptome annotation resulted in 26,589 transcripts matching 17,667 Uniprot-Swissprot entries (e-value < 10^−6^; Table 3) for a total of 11,985 transcripts associated with at least one of the Gene Ontology terms. For downstream expression analysis, only genes actually expressed in muscle (log CPM > 1 in at least two individuals) from the final assembly were retained, resulting in a total of 13,282 transcripts.

**Table 3:** Assembly and annotation statistics

Transcriptome statistics	
Total number contigs	33, 017
Percent GC	48.61
Contigs N50 (bp)	2,804
Total assembled bases	63, 545, 739
Median contig length (bp)	1,482
Average contig length (bp)	1,924
**Annotation**	
Contig with Uniprot-sp match (e-value 10^−6^)	26,589
Contig with GO identifier annotation	25,837

GO = gene ontology.

### Gene expression effects associated with domestication

We found that 206 genes were differentially regulated between wild and first-generation domesticated *C. auratus* (FDR < 0.01; |logFC| > 2), with 150 genes being up- and 56 down-regulated in wild individuals (Table 4). Genes differentially expressed between genotypes may include the effects of human artificial selection, founder effects, genetic drift and inadvertent selection due to the new rearing environment or to the selection of traits correlated with the traits of interest. In addition, it should also be noted that differences in the environment of domesticated and wild fish during the larval and juvenile phase could have affected long-term gene expression, and that some of these changes may have persisted even though fish were acclimatised to hatchery conditions for over 10 months before the trial started. The gene ontology analysis revealed that the most enriched GO terms were involved in strong global defense response (GO:0006952) and immune response (GO:0006955) (Supplementary Table 2). Interestingly, among the most strongly differentially expressed genes, we found that two serum amyloid proteins (A-1 and A-3) were heavily down-regulated in the F_1_-domestic *C. auratus* fish. Proteins or mRNAs of the highly conserved serum amyloid A family have been identified in all vertebrates investigated to date and function as major acute phase proteins in the inflammatory response (Uhlar and Whitehead 1999). The co-expression network analysis identified a single module with a highly significant genotype correlation (|R| > 0.75; *P* < 0.001), namely the darkorange2 module (R = 0.88). This module contained 94 genes and showed no significant enrichment, but tendencies for increased glutathione metabolic processes (GO:0004364) and transferase activity, transferring alkyl or aryl (other than methyl) groups (GO:0016765). Furthermore, the most negatively correlated module with genotype was the antiquewhite2 module (n = 1,227; R = −0.69), which showed enrichment for adaptive and innate immune response functions (GO:0002250 and GO:0045087, respectively), defense responses (GO:0006952) and a positive regulation of the Mitogen-Activated Protein Kinase (MAPK) cascade (GO:0043410) and ELK3 coding gene (antiquewhite2 module).

**Table 4:** Number of differentially expressed genes using a contrast approach and GLM model in edgeR. Genes were considered differentially expressed when FDR was below 1% and |log2FC| > 2. A single gene showed significant interaction (genotype x temperature; FDR < 1%)

Effect	Reference condition	Down-regulated	Up-regulated	Total
**Major effect**				
Temperature	Low	736	725	1,461
Genotype	Wild	56	150	206
**Interaction effect**				
Interaction Genotype x Temperature				1

In fish, the domestication process has been shown to influence metabolism, behavior and chronic stress and immune responses (Álvarez and Nicieza 2003; Millot *et al.* 2009; Douxfils *et al.* 2011a 2015). In the first fish study on this topic, Roberge *et al.* (2005) showed that juvenile Atlantic salmon (*Salmo salar*) had gene expression differences for at least 1.4 and 1.7% of the expressed genes, following 5 and 7 generations of domestication, respectively. Of the differentially expressed genes they found a general reduction in basal metabolic rate and an increased metabolic efficiency in farmed juvenile salmon, compared with its wild counterpart, favoring allocation of resources toward growth and fat deposition. This finding is consistent with the faster growth and higher fat yield in domesticated salmon from the same source (Rye and Gjerde 1996). Interestingly, they also detected two genes coding for MHC antigens that were more highly expressed in some farmed salmon, presumably in response to selection for disease resistance. In perch (*Perca fluviatilis)*, domestication increased the immune response during a challenge experiment with *Aeromonas hydrophila*, with a congruent increase in the levels of circulating HSP70 between the first and fourth generation in captivity (Douxfils *et al.* 2011a, 2011b). With respect to somatic growth, domestication responses in Coho salmon (*Oncorhynchus kisutch*) closely resemble responses seen following growth hormone (GH) gene insertion in other fish species, most likely due to the strong selection for enhanced growth rate (Devlin *et al.* 2009). Indeed, recent studies on GH transgenic salmon showed that the growth advantage resulting from GH transgenesis was tightly linked with and dependent on the immune system response capacity, suggesting that the GH/insulin-like growth factor (IGF) pathway interacts with global immune response pathways (Alzaid *et al.* 2018).

Results from the current study were similar to the observations reported in the studies described above, whereby differences in growth between domesticated and wild *C. auratus* occur through an interaction of the immune response and anabolic growth pathway modulation. Highlighted by the enrichment of immunity-related processes and the increased expression of the MAPK/ERK cascade — a key regulator of IGF-I- and IGF-II-mediated myogenesis and somatic growth regulation in both mammals and teleosts (Codina *et al.* 2008; Fuentes *et al.* 2011) — these interactions suggest that in the wild *C. auratus* immune-related activity was being prioritized over growth-related functions. This appeared to have negative consequences for the mass gain of the individuals over the experimental period. It is also noteworthy that modules segregating between genotypes were also tightly correlated with hematological indicators (Hb and MCHC outcomes, Table 1). When combined with the observation of green liver syndrome in the domestic low temperature treatment (results not presented), this outcome was considered to arise from a nutritional taurine deficiency in the experimental diets (Takagi *et al.* 2006; Matsunari *et al.* 2008), apparently exasperated by cold temperature exposure. It is noteworthy that this nutritional inadequacy interacts positively with the module enriched for immune response, and was not detected in wild individuals with the same recent nutritional history (*i.e.*, the same hatchery diet). This observation highlights the challenges of sourcing nutritionally adequate feed for non-model and pre-commercial cultured fish species, as well as the unknowns implicitly associated with investigations involving wild-caught fish. Additional studies will help to elucidate whether the patterns we observed result from different life history traits between genotypes (*e.g.*, exercise, contact with pathogens, acclimation to captivity, different environmental and nutritional conditions in early life) and/or are the result of relaxed or novel selection in the early domestication processes.

### Temperature had a major effect on gene expression

**Figure 2:**
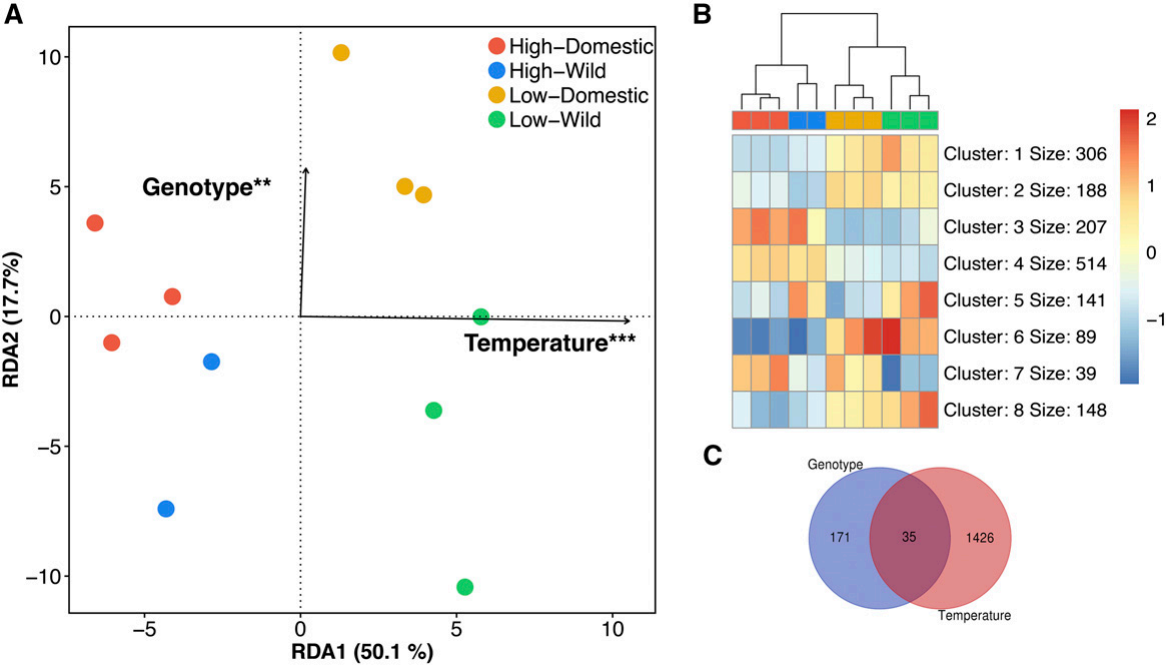
Effect of temperature and genotype one gene expression. A) Distance-base redundancy analysis (db-RDA) performed on the expression data (logCPM [prior count 2)]. Only genes with min logCPM > 1 in at least three samples were retained for the analysis (n = 14,372). The db-RDA model was globally significant (*P* < 0.001) and explained 59.8% of all expression variation (adj. *R*^2^ = 0.598). Genotype and temperature significantly explained 17.7% and 50.1% of the variation, respectively, after controlling for each other with subsequent partial db-RDAs. Significance codes: p-value < 0.001 ‘***’; p-value < 0.01 ‘**’; B) Heatmap and K-means clustering of genes showing differential expression between genotypes and/or temperature; C) Venn diagram showing the overlap between genotype and temperature in an additive effect (parallel reaction norms).

**Figure 3:**
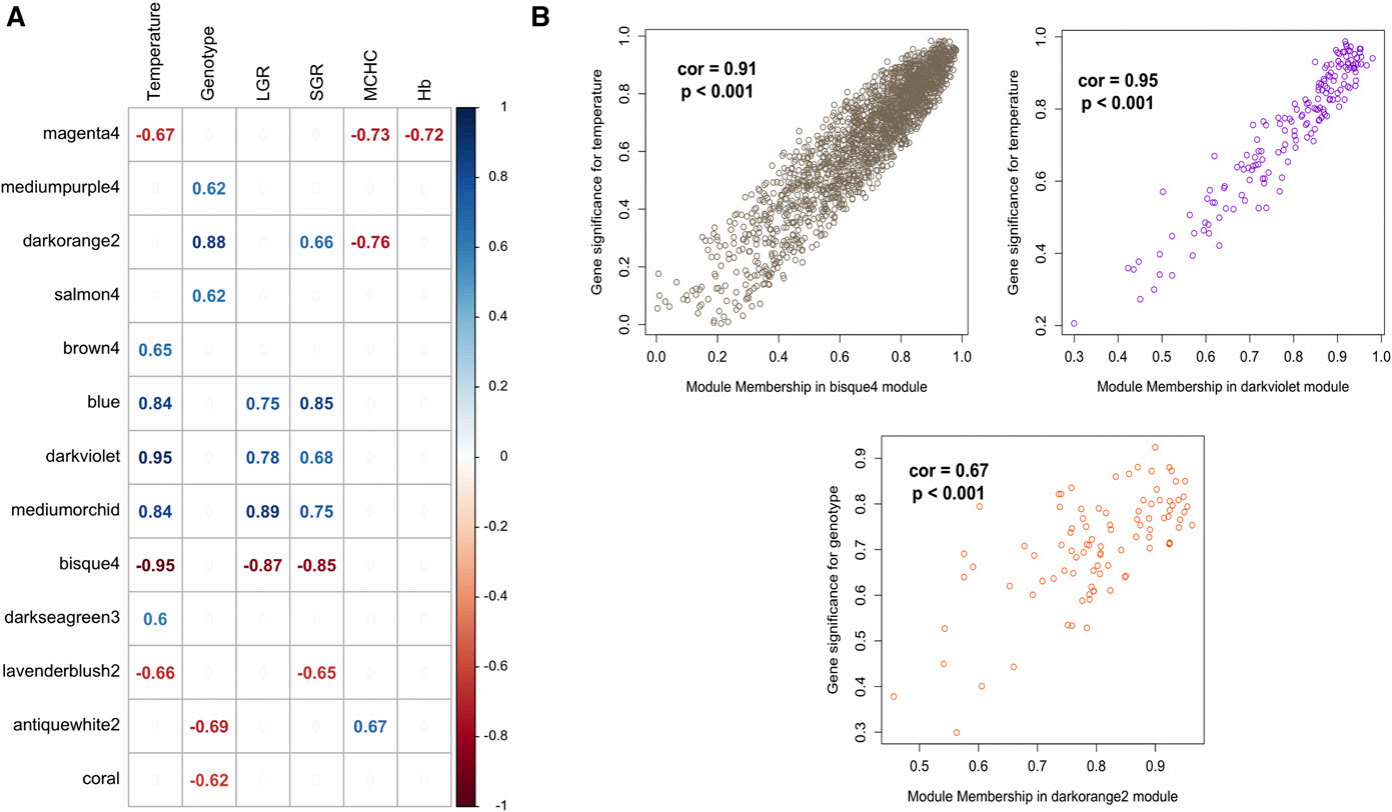
Co-expression network analysis. A) Correlation matrix from weighted gene co-expression network analysis (WGCNA). The matrix of co-expression was built on a total of 11,426 genes after removal of low-expressed genes (logCPM < 1 in at least two individuals and genes with gene expression variance < 0.1 in the global dataset). Only modules significantly correlating (p-value < 0.01) with temperature or genotype are represented. Values indicate the correlation value (R). B) Correlation between module membership and gene significance for the modules with the highest correlation to temperature (modules bisque4 and darkviolet) and genotype (module darkorange2).

To quantify the extent of gene expression variation associated with temperature and identify differentially expressed genes, we used a combined multivariate analysis (Redundant Discriminant Analysis; RDA) and GLM approach with an additive effect design (*e.g.*, by removing the gene in the interaction, see Methods for details). Overall, temperature had the most profound effect on the variation in expression, explaining 47.2% of the total variance (Figure 2). This corroborates the differential expression analysis whereby (despite stringent filters FDR < 0.01; |logFC| > 2) we found a large number of genes (n = 1,461) differentially regulated between the high and low temperature treatments, with 736 up- and 725 down-regulated genes in the high temperature relative to low temperature condition (Table 4; Figure 3). The gene ontology analysis on the total differentially expressed genes revealed that most enriched GO terms included tRNA aminoacylation for protein translation (GO:0006418) and sister chromatid segregation (GO:0000819), suggesting important differences in protein synthesis and cellular multiplication, both of which are key processes in myogenesis and somatic growth in fish.

We further investigated the global response to temperature using a co-expression network analysis, which has been shown to be particularly relevant for the functional analysis of non-model fish species (*e.g.*, Filteau *et al.* 2013). We identified a total of 37 modules associated with a temperature response, but chose to focus only on 13 of these modules, based on whether they were significantly correlated (*P* < 0.01) with either temperature and/or genotype (Figure 3). A total of four modules showed a highly significant correlation (|R| >0.75; p-value < 0.001) with temperature, with three being positively [blue (0.84), darkviolet (0.95), mediumorchid (0.84)] and one being negatively correlated with temperature [bisque4 (-0.95)]. Four other modules, the magenta4, brown4, darkseagreen3 and lavenderblush2, showed a significant (*P* < 0.05) yet lower correlation with temperature, (R = −0.67, 0.65, 0.66 and −0.66, respectively). We validated the level of association by computing the mean gene significance value for each module and found that most correlated modules showed the highest absolute mean gene significance to temperature (Figure 3; Supplementary Table 2). We also found that 81.4% of genes differentially expressed between temperature treatments were for the four most strongly correlated modules in the WGCNA analysis, which was a finding consistent with our network construction. Finally, a gene ontology enrichment analysis was conducted for each module (gene ontology enrichment results are compiled in Supplementary Table 2).

We went on to investigate the role of genes in the co-expression network in the global transcriptomic response by identifying several major genes associated with rapid growth, thermal compensation and/or a post-acclimation response. The network co-expression analysis revealed that the four modules that were most responsive to temperature (blue, darkviolet, mediumorchid and bisque4) were also strongly correlated with specific growth rate (SGR) and linear growth rate (LGR) traits (Table 1), suggesting that these modules are either directly involved or linked to growth modulation, independently of the genotype. For the blue module (n = 1,404 genes) we again found significant enrichment (p-adj < 0.05) for amino acid activation (GO:0043038) and (GO:0043039) in accordance with our results for the differential gene expression analysis, but also for the generation of precursor metabolites and energy (GO:0006091), oxidoreduction coenzyme metabolism (GO:006733) and electron chain transport (GO:0022900). For the darkviolet module (n= 169 genes), we found significant enrichment for the hemoglobin complex (GO:0005833) and oxygen transport activity (GO:0005344). For the mediumorchid module (n = 1,370 genes), we found significant enrichment for global cell adhesion and metabolism, including extracellular structure organization (GO:0043062), collagen metabolic process (GO:0032963) and multicellular organism metabolic process (GO:0044236). Finally, the bisque4 module (n = 2,063 genes) showed enrichment for functions involved in peroxisome structure and activity including protein import into peroxisome matrix, docking (GO:0016560) and peroxisomal membrane (GO:0005778). We also found that the module brown4 (n = 93 genes) had enrichment for protein folding (GO:0006457) and negative regulation of transcription from RNA polymerase II promoter in response to stress (GO:0097201), as well as a tendency for a response to heat function (GO:0009408; p-value < 0.001; p-adj = 1). Gene ontology enrichment results are compiled in Supplementary Table 2.

We also identified hub genes, which are reported as core regulating genes, within the most relevant four modules correlated with temperature based on their modular membership (kME) values (Figure 3). The hub genes selection identified key actors in the global temperature response within each module, often known as key regulators on biological pathways. Among the blue module, we identified several tRNA ligases but also the YTH domain-containing family protein 2 and the eukaryotic translation elongation factor 1 epsilon-1 that play a role in RNA protection following heat stress and DNA protection after damage, respectively (Lewis *et al.* 2017; Chen *et al.* 2018). Among the mediumorchid module we found cyclic AMP-dependent transcription factors (namely ATF-4 and -5) that are transcription factors associated with circadian rhythm regulation (Supplementary Figure S1) as well as the cryptochrome-1 coding gene, a core repressor of circadian rhythm (Hardie *et al.* 2012).

Given this extensive temperature-induced transcription-level biological reorganization, we focus the following interpretation on the most relevant results that corroborate previous transcriptional and physiological studies relating to the thermal responses of fish. HSP-coding genes (HSPs90 and HSPs70 and HSP-binding70) were among the highest levels for differentially expressed genes we detected. Mostly clustered within the brown4 module (R = 0.65 with temperature), a significant increase in HSP expression was observed in *C. auratus* at temperatures at both ends of the species thermal envelope — at least in the geographic location the fish were cultured or captured in. This HSP response is well known in fish as a stress-induced response, which functions to protect against oxidative stress and apoptosis (Lindquist and Craig 1988; Iwama *et al.* 2004; Oksala *et al.* 2014). HSP expression is often upregulated during short term (acute) exposures to high temperatures, as seen in the gill and muscle tissues of wild goby *Gillichthys mirabilis* exposed to 32° for up to 8 h (Buckley *et al.* 2006; Logan and Somero 2010). In addition, HSP upregulation can also be commonly observed during seasonally and environmentally relevant thermal regime shifts within the zone of sub-lethal thermal tolerance for a species (Fader *et al.* 1994; Oksala *et al.* 2014). The HSP response observed in this study, clustered within the brown4 module, showed no significant correlation with growth (both SGR and LGR), nor were there detectable differences between wild and domestic strains. This is notable as differences in HSP expression commonly underlie phenotypic differences within a species, and this has often been observed across geographical gradients (Fangue *et al.* 2006; Hirayama *et al.* 2006).

Temperature produced a pronounced phenotypic effect characterized by a positive change to the growth rate at warm temperature (∼21°), and negligible growth changes at low (∼13°) temperature. These phenotypic effects were underlined by substantial biological reorganization associated with metabolic fuel switching and a shift from anabolic metabolism at high temperatures to maintenance/catabolism at low temperatures (Figure S1). Notable upregulation of AMPK (mediumpurple4 module) was evident at low temperature. Commonly referred to as the cellular ‘master switch’, this signaler is known to produce a cascade of changes to cellular homeostasis to reduce energetically expensive metabolic pathways (Hardie 2007). The increased expression of driver genes within the PI3K-AKT-mTOR pathway (darkviolet module) clearly underscores the up-regulation of cellular signaling pathways and growth-related processes (*i.e.*, protein, lipid, and glycogen synthesis; cellular proliferation) at high temperature, all of which are known responses in fish (Fuentes *et al.* 2011, 2013). The upregulated PI3K-AKT activity together with the expression of FOXO1 (expressed within the bisque4 module) coding gene corroborates the reported atrophic/catabolic processes that we observed during the low temperature conditions. Notably, these growth responses were also associated with increased expression of the atrogin-1 muscle growth inhibitor and the downregulation of the muscle growth promoters insulin-like growth factor binding protein (IGFBP)-1 and -7 (also contained in the bisque4 module) (Glass 2005; Fuentes *et al.* 2013). The observed switch from catabolic to anabolic states strongly suggests a switching of how the metabolic energy was being used in *C. auratus* at the two different temperatures. The most down-regulated gene at high temperature was the long-chain fatty acid (LFA) transport protein 1, a gene involved in regulating LFA substrates in tissues undergoing high levels of beta-oxidation or triglycerides synthesis. The modulation of these transport processes corresponds with significant differences in circulating triglyceride levels (Table 1). Also associated with this process is the apparent ‘glucose sparing’ response and concomitant switch from carbohydrate- to lipid-based metabolism, inferred from the upregulation of both beta-oxidation by ACC2 and gluconeogenic pathways via the PEPCK and phosphofructokinase / fructose bisphosphate mediated metabolic pathways. Similarly, down-regulation of N-terminal glutamine aminohydrolase — a hub gene of the mediumorchid pathway that favors the production of glutamate — was observed in the high temperature treatment. It is only recently that glutamate has been shown to present a major non-carbohydrate-based energy substrate for skeletal muscle in fish (Weber *et al.* 2016; Jia *et al.* 2017).

The extensive reorganization of *C. auratus* metabolism across its natural temperature range presents an interesting area of future research, particularly with consideration of the vast seasonally-dependent growth differences evident. Moreover, the molecular mechanisms underlying the notable thermal plasticity present in many fishes are still poorly understood. Differential expression of genes has been investigated as a cause for phenotypic plasticity in three-spined stickleback (*Gasterosteus aculeatus)* (Metzger and Schulte 2018). Being affected by both developmental temperature and adult acclimation temperature, there are probable mechanistic links between gene transcription, epigenetic signatures and thermal plasticity across different time scales (Metzger and Schulte 2018). The plasticity in fish response to temperature may promote phenotypic alterations and ultimately, population divergence (Schulte 2014) during successive generations in both aquaculture and ecological contexts (Anttila *et al.* 2013; Donelson *et al.* 2018).

### Parallel and non-parallel thermal reaction norms

**Figure 4:**
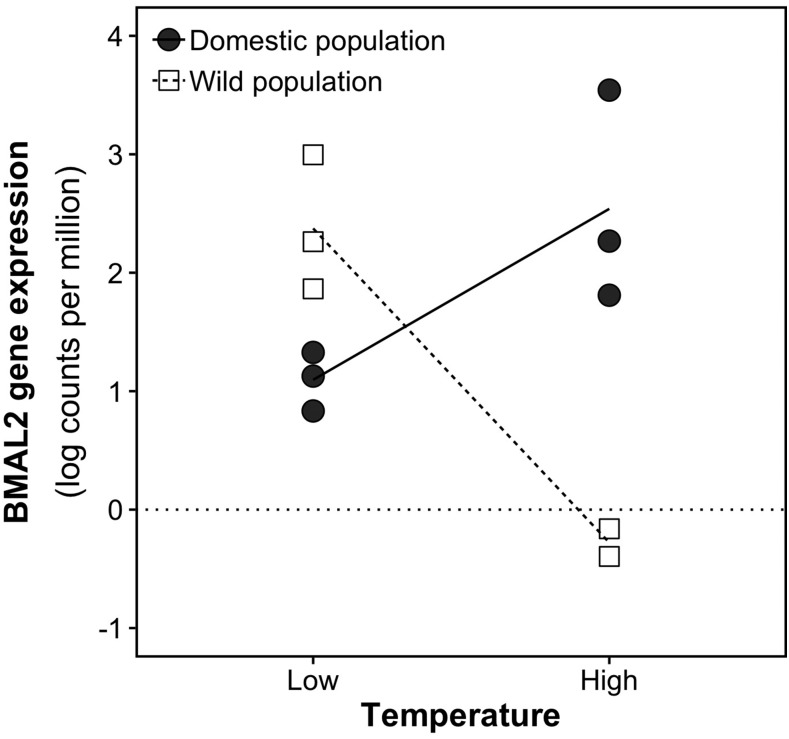
Interactions between temperature and genotype for BMAL2 gene expression.

A total of 35 genes showed parallel reaction norms whereby both wild and domesticated fish showed the same gene expression responses to temperature (*i.e.*, significant effects of temperature and genotype; Figure 2). We further tested the hypothesis that temperature may impact gene expression but to a different extent according to the genotype (interaction between temperature and genotype effects in a non-additive fashion; *i.e.*, non-parallel reaction norms). To detect Genotype by Environment Interactions (GEI), we used a GLM approach and a likelihood ratio test implemented in edgeR using the normalized data (Robinson *et al.* 2010). Only one gene, the aryl hydrocarbon receptor nuclear translocator-like protein 2 (Bmal2), showed a significant GEI (FDR < 0.01; Figure 4). The transcriptional activator BMAL2 is a core component of circadian rhythm regulation in mammals (Ikeda *et al.* 2000). Temperature-dependent activation and compensation of circadian rhythm have been observed in both vertebrates and invertebrates (Menaker and Wisner 1983; Sawyer *et al.* 1997; Rensing and Ruoff 2002; Zhdanova and Reebs 2006). Furthermore, different modulation of the circadian rhythm suggested adaptation to environmental cues after selective breeding for growth-related traits during the early domestication process (López-Maury *et al.* 2008). Similarly, a switch in behavior (day or nocturnal activity) has been observed during both temperature experiments and domestication selection in European sea bass (*Dicentrarchus labrax*) (Millot *et al.* 2009, 2010). The contrasting responses to temperature depending on the genotype background for some of the core regulators suggest that selection for a specific trait in aquaculture is also dependent on the rearing environment. More studies will be required to tease apart the responses to selection from any domestic environment-induced or plasticity effects that could occur during the larval rearing phase. Nevertheless, plasticity has significant impacts on the genetic gain calculation in many livestock breeding programs (Mulder 2016; Nguyen *et al.* 2017), and will be an important parameter to assess for newly domesticated species, including fish.

## Conclusions

Although fish domestication has received considerable interest for many years from a range of disciplines, modern large-scale genomic technologies and newly formed captive populations provide a unique opportunity to shed light on an important and well-documented evolutionary change for aquatic species. In this study, we combined GLM-based approaches to investigate synergistic effects of recent domestication and temperature effects on gene expression regulation in the Australasian *C. auratus*. Coupling differential expression clustering and gene co-expression networks allowed us to begin to untangle the complex mechanisms of growth modulation during the first steps of selection and acclimation to domestication conditions. Our study shows that recent domestication and temperature had combined effects on muscle gene expression levels. We observed that temperature affected primarily HSP responses as well as tissue development and cell turnover while genotype mainly affected the global immune response. Only a single gene (*Baml2*), crucial for circadian rhythm control, was affected by GEI. Admittedly, the present study only assayed a single tissue and further investigation of brain or hormone-producing tissue would produce a better understanding of the role of behavioral changes and immune responses that occur during the first few generations of domestication selection. Our study adds to the small number of previous studies that showed that gene expression responses can change rapidly following a few generations of domestication.
